# Low-Frequency Whole-Body Electromyostimulation (WB-EMS) for Nonspecific Chronic Back Pain: A Systematic Review and Meta-Analysis

**DOI:** 10.7759/cureus.88462

**Published:** 2025-07-21

**Authors:** Karl L Konrad, Patrick Sadoghi, Jana B Konrad, Erik Cattrysse, Jean-Pierre Baeyens, Bernd Wegener

**Affiliations:** 1 EXAN (Experimental Anatomy) Research Group, Department of Physiotherapy, Human Physiology, and Anatomy, Vrije Universiteit Brussel (VUB), Brussels, BEL; 2 Department of Therapy Research, Institut Ascend, Röfingen, DEU; 3 Department of Orthopaedic Surgery, Physical Medicine, and Rehabilitation, Ludwig-Maximilians-University (LMU), Munich, DEU; 4 Department of Orthopaedics and Traumatology, Medical University Graz, Graz, AUT; 5 Department of Therapy Research, THIM University College Physiotherapy, Landquart, CHE

**Keywords:** back pain, electric stimulation therapy, electrotherapy, exercise therapy, functional improvement, neuromuscular electrical stimulation (nmes), nonspecific chronic back pain (nscbp), pain intensity, pain measurement, whole-body electromyostimulation (wb-ems)

## Abstract

This systematic review aims to evaluate the effectiveness of low-frequency (LF) whole-body electromyostimulation (WB-EMS) in reducing pain and improving function in patients with nonspecific chronic back pain (NSCBP). Given the global prevalence of NSCBP and the limitations of conventional exercise due to time constraints and comorbidities, LF WB-EMS, a time-efficient and joint-friendly intervention, has emerged as a potential alternative. All prior PubMed studies on WB-EMS and back pain have exclusively investigated LF-EMS (0-999 Hz), and this review assesses its efficacy compared to a passive control group (PCG) or active control group (ACG), addressing a gap in understanding its clinical utility for NSCBP management.

Following Preferred Reporting Items for Systematic Reviews and Meta-Analyses (PRISMA) guidelines, PubMed and Physiotherapy Evidence Database (PEDro) were systematically searched for clinical trials from database inception to the present using inclusion criteria encompassing adults with NSCBP (pain >12 weeks, no specific cause), WB-EMS interventions, and randomized controlled trials (RCTs) or controlled clinical trials (CCTs) reporting pain and function outcomes. Data were extracted by two independent reviewers using a standardized form, and quality was assessed with the PEDro Scale. A narrative synthesis described study characteristics, while a meta-analysis using IBM SPSS Statistics software, version 22 (IBM Corp., Armonk, NY), employed an inverse variance-weighted method to pool standardized mean differences (SMD) with 95% confidence intervals, assessing heterogeneity with I². Studies lacking a distinct non-WB-EMS control group or with mixed control groups not clearly assignable to passive or active controls were excluded from comparative analysis but included in within-group analysis where appropriate. Six studies (n = 677, WB-EMS: 278, controls: 329) were included, comprising four RCTs, one CCT, and one meta-analysis (2017-2023). LF WB-EMS (20-minute sessions, once a week, eight to 16 weeks, 50-85 Hz) significantly reduced pain (-0.60 to -1.58 Numeric Rating Scale (NRS)/Visual Analog Scale (VAS)) and improved function (+7.19 kg to -15.8 Oswestry Disability Index (ODI)) within groups. Meta-analysis of five studies showed a pooled pain reduction of -0.87 (95% CI (-1.02, -0.72), I² = 70%) and functional SMD of 0.84 (95% CI (0.68, 0.99), I² = 76%). Against passive controls (n = 15/group), effect sizes were 0.75 (pain) and 0.85 (function), while versus active controls, pooled effects were 0.33 (pain, I² = 96%) and 0.28 (function, I² = 92%), with high heterogeneity.

The results indicate that LF WB-EMS can reduce pain and improve function in NSCBP, with within-group effects of -0.87 NRS and 0.84 SMD and comparative effects of 0.75/0.85 vs. PCG and 0.33/0.28 vs. ACG, indicating potential benefits despite high heterogeneity and modest effect sizes. WB-EMS shows comparable efficacy to established methods, offering a promising option for patients with time or mobility constraints, supported by its safety and joint-friendly nature. However, limitations, including a small PCG sample (n=15) and limited research on medium-frequency WB-EMS, necessitate larger trials and further studies to optimize protocols and confirm long-term efficacy.

## Introduction and background

Chronic back pain predominantly affects individuals aged 40 to 69, with a higher prevalence among women, and is more common in high-income countries where sedentary lifestyles prevail [1]. In approximately 90% of cases, chronic back pain lacks a specific anatomical cause beyond normal spinal degeneration, classifying it as nonspecific [2]. Nonspecific chronic back pain (NSCBP) has emerged as a major global health challenge, being the most prevalent chronic condition worldwide and surpassing diabetes, cardiovascular disease, hypertension, respiratory illnesses, asthma, and cancer combined in its impact on workforce participation [3-4]. It significantly impairs quality of life and contributes to a considerable rise in disability-adjusted life years (DALYs), making it a leading cause of disability globally [5].

The development of NSCBP is influenced by multiple factors, including repetitive strain, imbalanced movement patterns, and physical inactivity, with sedentary lifestyles in developed countries strongly associated with its rising incidence [6-8]. A sedentary lifestyle often leads to reduced physical activity, which weakens muscle strength and power, particularly in the trunk muscles essential for spinal stability [9]. Consequently, physical exercise is widely regarded as the primary treatment for NSCBP, as supported by European and national guidelines [2,10]. Systematic reviews, analyzing 45 and 61 trials respectively, confirm that exercise programs focusing on strength, coordination, and stabilization yield superior results in pain reduction and functional improvement compared to other treatments [11,12]. Hayden et al. (2005) and Searle et al. (2015) reported consistent findings, showing better outcomes with such exercise programs [11,12].

Despite the established advantages of physical exercise, adherence among NSCBP patients remains low, often due to the demanding nature of modern life, which limits the time available for regular training [13]. The fast-paced lifestyle prevalent today creates a substantial obstacle for individuals attempting to overcome inactivity and engage in physical activity [14]. Additionally, comorbidities such as osteoarthritis further complicate the adoption of consistent exercise routines, posing a significant challenge to effective NSCBP management [15]. This low adherence highlights a critical gap in conventional therapies, where time constraints and joint issues reduce effectiveness, underscoring the need for alternatives like whole-body electromyostimulation (WB-EMS). In this context, WB-EMS has gained attention as a novel, time-efficient, and joint-friendly treatment option. WB-EMS simultaneously stimulates multiple muscle groups, including the trunk muscles, through electrical impulses while the individual performs voluntary movements. Its popularity in both fitness and rehabilitation settings is largely due to its ability to provide a comprehensive workout in a shorter time frame, making it attractive for those with busy schedules or physical limitations [16]. Due to its time efficiency, WB-EMS offers a promising option for individuals with a busy lifestyle despite time constraints, addressing a key barrier to conventional exercise in NSCBP management. Moreover, clinical trials in NSCBP patients have indicated that WB-EMS can significantly reduce pain, positioning it as a viable alternative to conventional exercise therapies [17-22]. Early research by Filipovic et al. (2012) established WB-EMS as an effective strength training method in athletes [23], while Herrero et al. (2006) demonstrated its comparative benefits over plyometric training in improving muscle performance [24], providing a foundation for its application in NSCBP. Its mechanism involves concurrent activation of agonists and antagonists, enhancing muscle contraction without external loads, which reduces joint stress and makes it suitable for patients with mobility limitations [23,24]. Numerous studies have demonstrated the positive effects of WB-EMS on muscle performance, showing improvements in strength and endurance across various muscle groups [23-32]. Previous clinical applications include sarcopenia management in elderly women, where WB-EMS improved body composition and strength with a favorable safety profile, reporting only mild muscle soreness as an adverse event [28]. To date, to the best of our knowledge, no PubMed-published systematic review specifically addressing WB-EMS for NSCBP exists, which is crucial for formulating future recommendations or integrating results into clinical guidelines (parts of this background on NSCBP and its management have been adapted from our previous work [33]).

Given the promising results of WB-EMS in other settings, this review aims to systematically assess the available clinical evidence on the effectiveness of WB-EMS in reducing pain and improving function in patients with nonspecific chronic back pain. Specifically, the objective of this review is to evaluate the outcomes of WB-EMS interventions alone and compared to conventional therapies or passive control in alleviating pain and enhancing physical function in individuals with NSCBP.

Varieties of EMS

**Figure 1:**
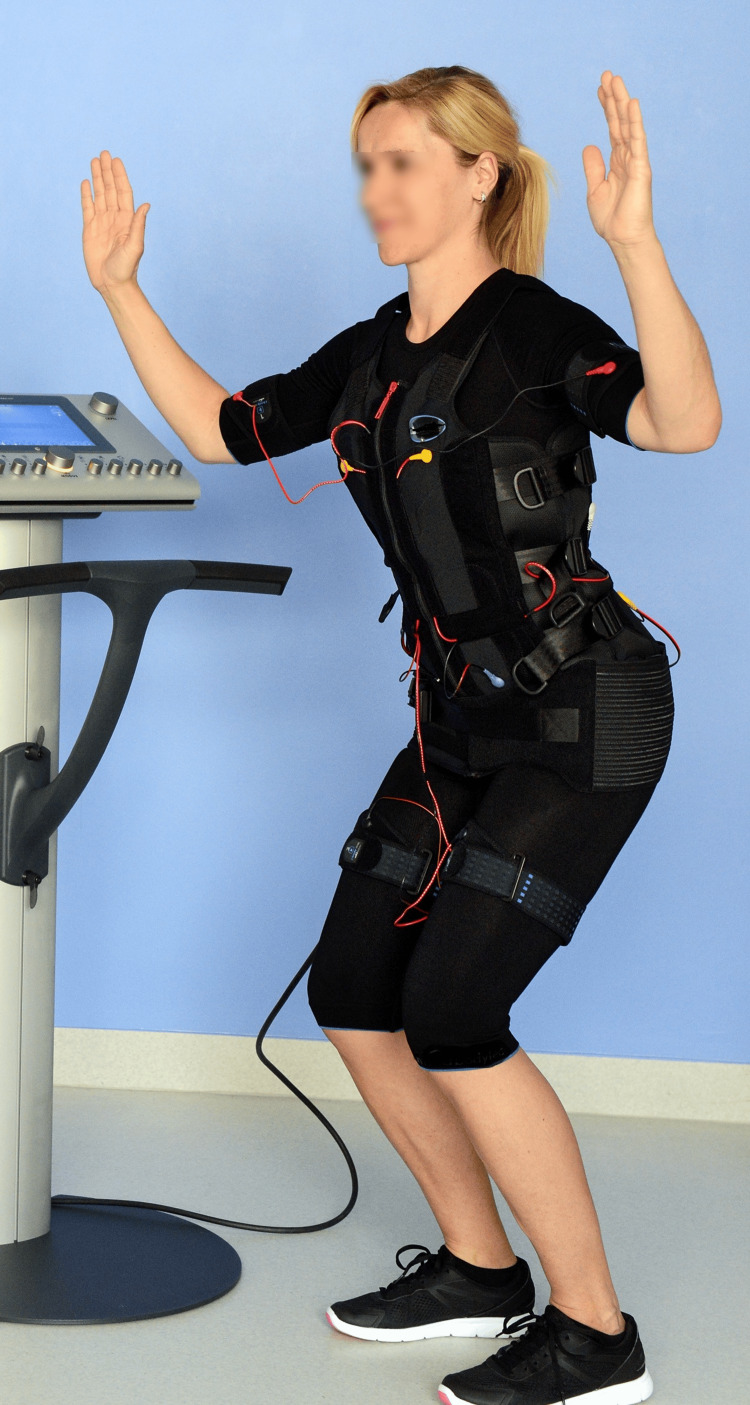
Typical WB-EMS therapy session A typical whole-body electromyostimulation (WB-EMS) therapy session, depicting a model wearing a vest and straps with electrodes that transmit electrical impulses to the body. Image credit: This image has been photographed by the research team working on this study.

WB-EMS is a form of electrical muscle stimulation (EMS), also known as neuromuscular electrical stimulation (NMES), which involves the simultaneous stimulation of large muscle groups, particularly the trunk and proximal extremities, during voluntary movements (Figure 1). Unlike local EMS, which targets isolated muscles, WB-EMS employs concurrent stimulation of agonists and antagonists, typically at low frequencies (LF) (LF-EMS, 0-999 Hz, mainly 20-90 Hz) or modulated medium frequencies (MF, 1-300 kHz, mainly 2-15 kHz), with LF-EMS associated with more rapid strength gains and MF-EMS linked to deeper, more physiological contractions [32]. All studies on WB-EMS and NSCBP published in PubMed to date have exclusively investigated LF-EMS, focusing on its efficacy in pain reduction and functional improvement. Accordingly, this study also examines LF-WB-EMS, applied concurrently with voluntary movement, to evaluate its effectiveness in managing NSCBP. LF-WB-EMS interventions typically consist of 20-minute therapist-guided sessions conducted once or twice per week, employing frequencies between 50 and 85 Hz, biphasic rectangular impulses with a pulse width of 350 µs, and an intensity set to a rate of perceived exertion (RPE) of five to seven.

## Review

Methods

Study Design

This systematic review was conducted to assess the current evidence on the effectiveness of WB-EMS in the management of NSCBP. The review follows the Preferred Reporting Items for Systematic Reviews and Meta-Analyses (PRISMA) guidelines [34] to ensure transparency and reproducibility throughout the process.

Data Sources and Search Strategy

The databases PubMed and Physiotherapy Evidence Database (PEDro) were systematically searched for relevant clinical studies. The search encompassed all clinical trials and meta-analyses published from the inception of these databases to the present, with a focus on English-language articles. The following search terms were used to identify relevant studies: ‘whole-body electromyostimulation’ OR ‘WB-EMS’ AND ‘back pain’ OR ‘chronic back pain’ OR ‘nonspecific back pain’. The exact search string used in PubMed, (("whole-body electromyostimulation") OR (WB-EMS)) AND ((back pain) OR (chronic back pain) OR (nonspecific back pain)), including Boolean operators, is provided in Appendix A to enhance transparency and enable replication. The search was conducted on January 16, 2025, ensuring inclusion of the most recent studies available at the time of analysis.

Study Selection

Two independent reviewers screened the titles and abstracts of identified studies for eligibility. Full-text versions of studies that met the inclusion criteria were retrieved and assessed for eligibility based on predefined inclusion and exclusion criteria. Any disagreements between reviewers were resolved through discussion or, if necessary, arbitration by a third reviewer.

Inclusion and Exclusion Criteria

Inclusion criteria: This review included studies involving adult participants aged 18 years and older who were diagnosed with NSCBP, defined as back pain lasting for more than 12 weeks without a specific pathological cause. The intervention of interest was WB-EMS. It was necessary that the studies included a comparator group, which could be either a control group receiving a placebo or no intervention, or an active comparator group receiving other physical therapies or exercise regimens. The primary outcomes of interest were pain intensity and functional improvement. Eligible study designs included randomized controlled trials (RCTs) and controlled clinical trials (CCTs).

Exclusion criteria: Studies that investigated specific back pain etiologies, such as disc herniation or spinal stenosis, were excluded. Studies utilizing local EMS were also excluded. Additionally, non-clinical studies, including reviews and case reports, were excluded from this review.

Data Extraction

A standardized data extraction form was used to collect key information from each included study. The following information was collected: study design, participant characteristics, intervention details, and outcomes (pain reduction, functional improvement, quality of life, and adverse effects) (Table 1). Two independent reviewers extracted the data, and any disagreements were resolved through consensus.

**Table 1:** Characteristics and outcomes of studies on WB-EMS for NSCBP Characteristics and outcomes of studies evaluating whole-body electromyostimulation (WB-EMS) effects on pain (Numeric Rating Scale (NRS), Visual Analog Scale (VAS)) and function (trunk strength, Oswestry Disability Index (ODI)) in nonspecific chronic back pain (NSCBP) patients [17-22]. Effect sizes (ES) are standardized mean differences. RCT: randomized controlled trial; CCT: controlled clinical trial; Non-RCT: non-randomized controlled trial; WBV: whole-body vibration; CT: conventional training; WBS: WB-EMS with stretching

Study	Study Design	Sample Size (WB-EMS, Control)	Pain Measure	WB-EMS Pain Delta (SD)	Control Pain Delta (SD)	Pain ES	Function Measure	WB-EMS Function Delta (SD)	Control Function Delta (SD)	Function ES	Time Point	Control/Comparator Intervention	PEDro Score
Weissenfels et al., 2018 [17]	RCT	15, 15	NRS avg	-0.74 (0.87)	-0.08 (0.88)	0.754	Trunk strength (kg)	+7.26 (9.69)	-1.03 (9.75)	0.853	12 weeks	Passive control (no intervention)	8/10
Weissenfels et al, 2019 [18]	RCT	55, 55	NRS avg	-0.60 (0.96)	-0.85 (0.97)	0.26	Trunk strength (kg)	+7.19 (8.82)	+8.96 (8.78)	0.2	12 weeks	Back-strengthening training (60 min, 1x/week, 12 weeks)	8/10
Konrad et al., 2020 [19]	CCT	85, 43	NRS	-1.58 (1.91)	0.66 (1.87)	0.51	ODI	-15.8 (11.77)	-0.88 (5.62)	0.51	12w / 4w	Multimodal treatment (whole Day, 5x/week, 4 weeks)	6/10
Micke et al., 2021 [20]	RCT (3-arm)	80, 80 (CT), 80 (WBV)	NRS	-0.91 (1.20)	CT: -0.95 (1.23); WBV: -0.89 (0.59)	CT: 0.02; WBV: 0.57	Trunk extension (kg)	+8.04 (11.99)	CT: +9.18 (11.69); WBV: +7.37 (4.08)	CT: 0.06; WBV: 0.39	12 weeks	WBV (20 min, 2x/week, 12 weeks); CT (60 min, 1x/week, 12 weeks)	8/10
Kemmler et al., 2017 [21]	Meta-analysis (RCTs)	23, 22	NRS	-0.87 (1.06)	0.00 (1.02)	0.84	Trunk strength (N)	N/A	N/A	0.72	12-16 weeks	Varies (passive or exercise)	7/10
Silvestri et al., 2023 [22]	Non-RCT	20, 20	VAS	-0.935 (0.69)	-2.76 (0.78)	-	ODI-I	-1.53 (1.24)	-7.42 (1.78)	-	8 weeks	WB-EMS alone (WBS: +6x30min stretching)	4/10

Quality Assessment

The quality of the included studies was evaluated using the PEDro Scale, a validated tool for assessing the methodological rigor of clinical trials [35], which has shown high correlation with the Cochrane Back and Neck (CBN) Group Risk of Bias Tool in evaluating trial quality [36]. The PEDro Scale assesses methodological quality across 11 criteria, with each criterion scored as yes (one point) or no (0 points). Criteria include eligibility criteria specified, random allocation, concealed allocation, baseline comparability, blinding of subjects, blinding of therapists, blinding of assessors, adequate follow-up (>85%), analyzed subjects getting full treatment or intention-to-treat analysis, between-group comparisons, and point estimates and variability. Total scores range from 0 to 10 (criterion one is not included in the total score) (Table 2). Scores ≥6 indicate high methodological quality, while scores <6 suggest moderate to low quality. Each study was rated by two independent reviewers, and any discrepancies were discussed and resolved by a third reviewer if necessary. Studies in which one of the authors of this review was involved were exclusively assessed by an independent researcher who has not been involved in the study. Studies with a PEDro score <5 were excluded from the meta-analysis due to a considerable risk of bias.

**Table 2:** PEDro quality assessment scores for included studies The PEDro Scale assesses methodological quality across 11 criteria (e.g., random allocation, blinding, follow-up), scored as yes (one point) or no (0 points). Total scores range from 0 to 10 (criterion 1 excluded). Scores ≥6 indicate high quality, four to five indicate moderate quality, and <4 show low quality [35]. PEDro: Physiotherapy Evidence Database; *: included trials in meta-analysis; AVG: average of included trials

Study	Eligibility	Random Allocation	Concealed Allocation	Baseline Comparability	Blinding Subjects	Blinding Therapists	Blinding Assessors	Adequate Follow-up	Intention-to-treat	Between-group Comparisons	Point Measures and Variability	Total Score
Weissenfels et al., 2019 [18]	Yes	Yes (1)	Yes (1)	Yes (1)	No (0)	No (0)	Yes (1)	Yes (1)	Yes (1)	Yes (1)	Yes (1)	8/10
Weissenfels et al., 2018 [17]	Yes	Yes (1)	Yes (1)	Yes (1)	No (0)	No (0)	Yes (1)	Yes (1)	Yes (1)	Yes (1)	Yes (1)	8/10
Konrad et al., 2020 [19]	Yes	No (0)	No (0)	Yes (1)	No (0)	No (0)	Yes (1)	Yes (1)	Yes (1)	Yes (1)	Yes (1)	6/10
Micke et al., 2021 [20]	Yes	Yes (1)	Yes (1)	Yes (1)	No (0)	No (0)	Yes (1)	Yes (1)	Yes (1)	Yes (1)	Yes (1)	8/10
Kemmler et al., 2017 [21]	Yes	Varies*	Varies*	Yes*	No*	No*	Yes*	Yes*	Yes*	Yes*	Yes*	7/10 (AVG)
Silvestri et al., 2023 [22]	Yes	No (0)	No (0)	Yes (1)	No (0)	No (0)	No (0)	Yes (1)	No (0)	Yes (1)	Yes (1)	4/10

Data Synthesis and Analysis

A narrative synthesis was conducted for all included studies to describe study characteristics, interventions, and outcomes qualitatively. Where data were sufficiently homogenous, a meta-analysis was performed using IBM SPSS Statistics software, version 22 (IBM Corp., Armonk, NY) [30]. Continuous outcomes, such as pain intensity and functional improvement, were reported as mean differences (MD) or standardized mean differences (SMD), calculated as the difference in means divided by the pooled standard deviation, along with 95% confidence intervals. The inverse variance-weighted method was employed to pool effect sizes, with variance calculated as $\frac{SD^2}{N}$, and weights as $\frac{1}{Variance}$. Pooled estimates were derived using $\frac{\sum (Weight \times Effect)}{\sum Weight}$, and standard errors (SE) were computed as $\frac{1}{\sqrt{\sum Weight}}$, with 95% CIs determined as $Effect \pm 1.96 \times SE$. Heterogeneity was assessed using the $I^2$ statistic, calculated as: $I^2 = \left( \frac{Q - df}{Q} \right) \times 100$ where $Q$ is the heterogeneity statistic and $df$ is the degrees of freedom. A random-effects model was applied if substantial heterogeneity was detected ($I^2 > 50\%$). Studies were excluded from the comparative meta-analysis (WB-EMS vs. passive control group (PCG)/active control group (ACG)) if no distinct control group without WB-EMS was present (e.g., comparing WB-EMS with WB-EMS + stretching, with only the WB-EMS group included in within-group analysis) or if the control group was mixed and could not be clearly assigned to PCG or ACG (e.g., with passive or exercise-based controls, with within-group data included).

Results

Study Selection

**Figure 2:**
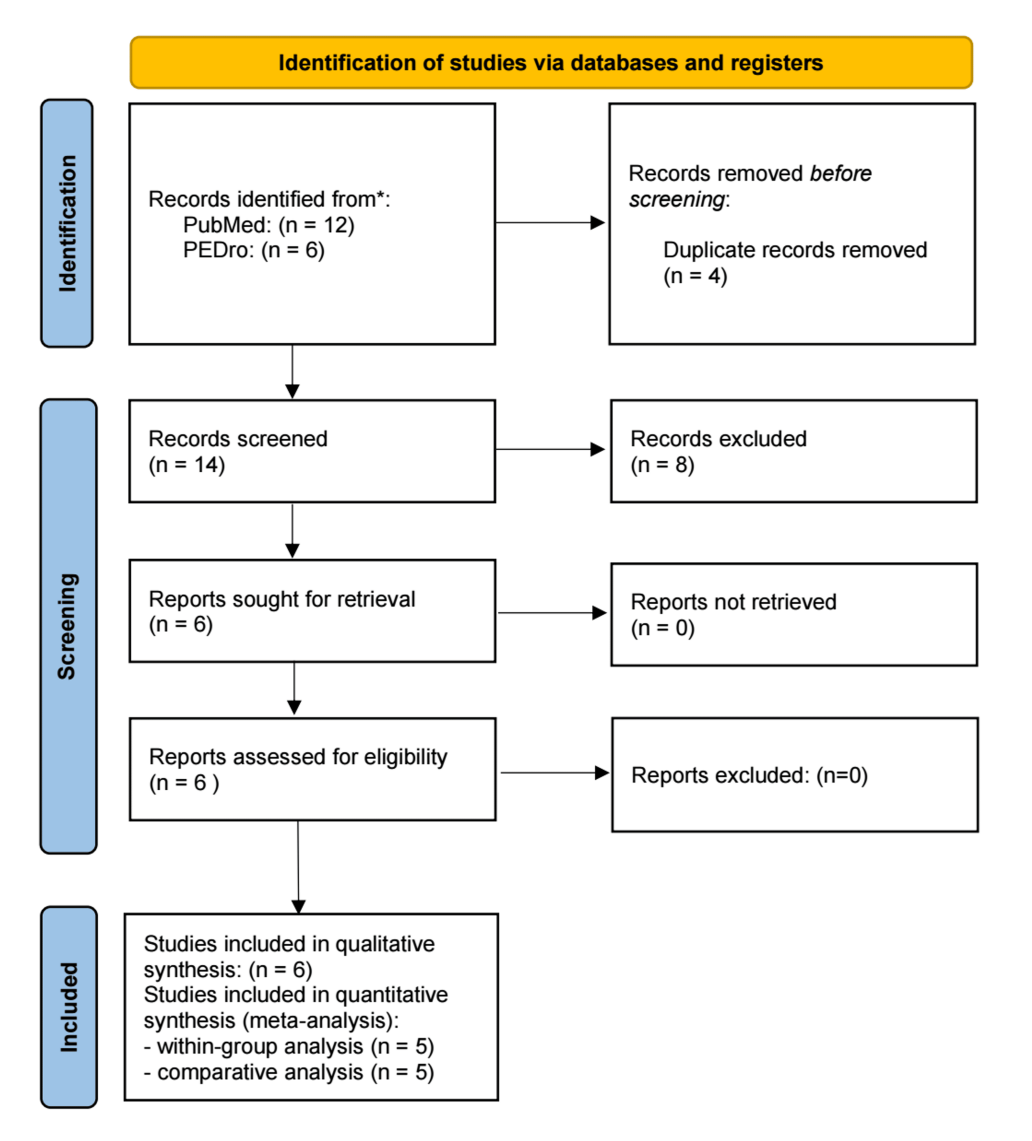
Study selection process for the systematic review, following the Preferred Reporting Items for Systematic Reviews and Meta-Analyses (PRISMA) guidelines Source: [34] PEDro: Physiotherapy Evidence Database

The systematic search identified 12 studies from PubMed and six studies from PEDro, resulting in a total of 18 records. After removing four duplicates, 14 unique records remained for screening. Following title and abstract screening, eight studies were excluded for not meeting the inclusion criteria. Consequently, six full-text articles were assessed for eligibility, all of which were included in the qualitative synthesis. Of these, five studies were deemed suitable for quantitative synthesis (meta-analysis), while one study was excluded from the comparative meta-analysis due to the absence of a control group without WB-EMS, though its WB-EMS group was included in the within-group analysis and qualitative synthesis (Figure 2) [17-22].

Narrative Synthesis

Study characteristics: The review included six studies published between 2017 and 2023 (Table 1): four RCTs, one CCT, and one meta-analysis of RCTs, with a total of 677 participants (WB-EMS: 278, control/comparator: 329) [17-22]. A non-randomized controlled study was also included [22]. Participant ages ranged from approximately 43 to 81 years, with a female predominance (61%-68%). WB-EMS interventions typically involved 20-minute sessions once weekly over eight to 16 weeks, using frequencies of 50-85 Hz and a pulse width of 350 µs, with intensity adjusted to an RPE of five to seven, except in one study, where frequency progressed from 50 Hz to 85 Hz [22]. Comparators varied: one study used a passive control (no intervention), another employed back-strengthening training (60 min, once per week), a third applied multimodal treatment (whole day, five times per week, four weeks), and a fourth compared WB-EMS to whole-body vibration (WBV; 20 min, two times per week) and conventional training (CT; 60 min, once per week) [17-20]. The meta-analysis pooled data from studies with mixed passive or exercise-based controls [21], while Silvestri et al. compared WB-EMS alone with WB-EMS plus six 30-minute stretching sessions [22].

Quality Assessment

The quality of the included studies was evaluated using the PEDro Scale, which assesses methodological quality across 11 criteria, with scores ranging from 0 to 10 (criterion one excluded). PEDro scores ranged from four to eight [17-22]. Three RCTs scored 8/10, with strong randomization [17,18,20], while the meta-analysis averaged 7/10 [21]. The CCT of Konrad et al. scored 6/10 due to non-randomization [19]. The non-randomized study by Silvestri et al. (2023) scored 4/10 [22] and was therefore excluded from the meta-analysis due to high risk of bias (Table 2). Scores ≥6 indicate high quality, four to five moderate, and <4 low quality [35].

Pain and Function Within Groups

All five studies reported significant within-group improvements in pain and function with WB-EMS [17-21]. Pain reductions ranged from -0.60 Numeric Rating Scale (NRS) points (SD=0.96) to -1.58 NRS points (SD=1.91), while functional improvements ranged from +7.19 kg (SD=8.82) trunk strength to -15.8 Oswestry Disability Index (ODI) (SD=11.77) [17-21]. The meta-analysis study reported a pain reduction of -0.87 NRS points (SD=1.06), with no functional data provided [21]. These improvements are supported by the meta-analytic pooled estimates, which showed a pain reduction of -0.87 NRS points (95% CI (-1.02, -0.72)) and a functional standardized mean difference (SMD) of 0.84 (95% CI (0.68, 0.99)). The moderate heterogeneity in the within-group analysis (I² = 70% for pain, I² = 69% for function) suggests variability across studies, which may be attributed to differences in intervention protocols (e.g., duration, frequency, intensity), participant characteristics (e.g., age, baseline pain severity), and outcome measures (e.g., NRS, Visual Analog Scale (VAS), ODI, trunk strength).

Comparative Effects

WB-EMS vs. PCG: One study showed WB-EMS reduced pain by -0.74 NRS (n=15) points versus -0.08 (SD=0.88) in the passive control (n=15), and trunk strength increased by +7.26 kg versus -1.03 kg (SD=9.75) [17].

WB-EMS vs. ACG: Another study reported -0.60 NRS points versus -0.85 (SD=0.97) with back-strengthening training and +7.19 kg versus +8.96 kg (SD=8.78) [18]. A third found -1.58 NRS points versus +0.66 (SD=1.87) with multimodal treatment and -15.8 ODI versus -0.88 (SD = 5.62) [19]. A fourth noted -0.91 NRS points versus -0.95 (CT, SD=1.23) and -0.89 (WBV, SD=0.59), with +8.04 kg versus +9.18 kg (CT, SD=11.69) and +7.37 kg (WBV, SD=4.08) [20]. These findings indicate varied effects across active controls, with pooled effects suggesting competitive performance [18-20].

Safety and Adverse Events

Adverse events were minimal, with mild muscle soreness in one to five participants, supporting WB-EMS's safety [17-22].

Limitations and Heterogeneity

Varied durations (eight to 16 weeks), diverse outcomes (NRS, VAS, ODI, trunk strength), and comparator heterogeneity (passive vs. active) pose challenges [17-22]. Lack of blinding in some studies and non-randomized designs may bias results, while small samples (e.g., n=30) and dropouts (e.g., 23%) limit generalizability [17,22].

Meta-Analysis

**Figure 3:**
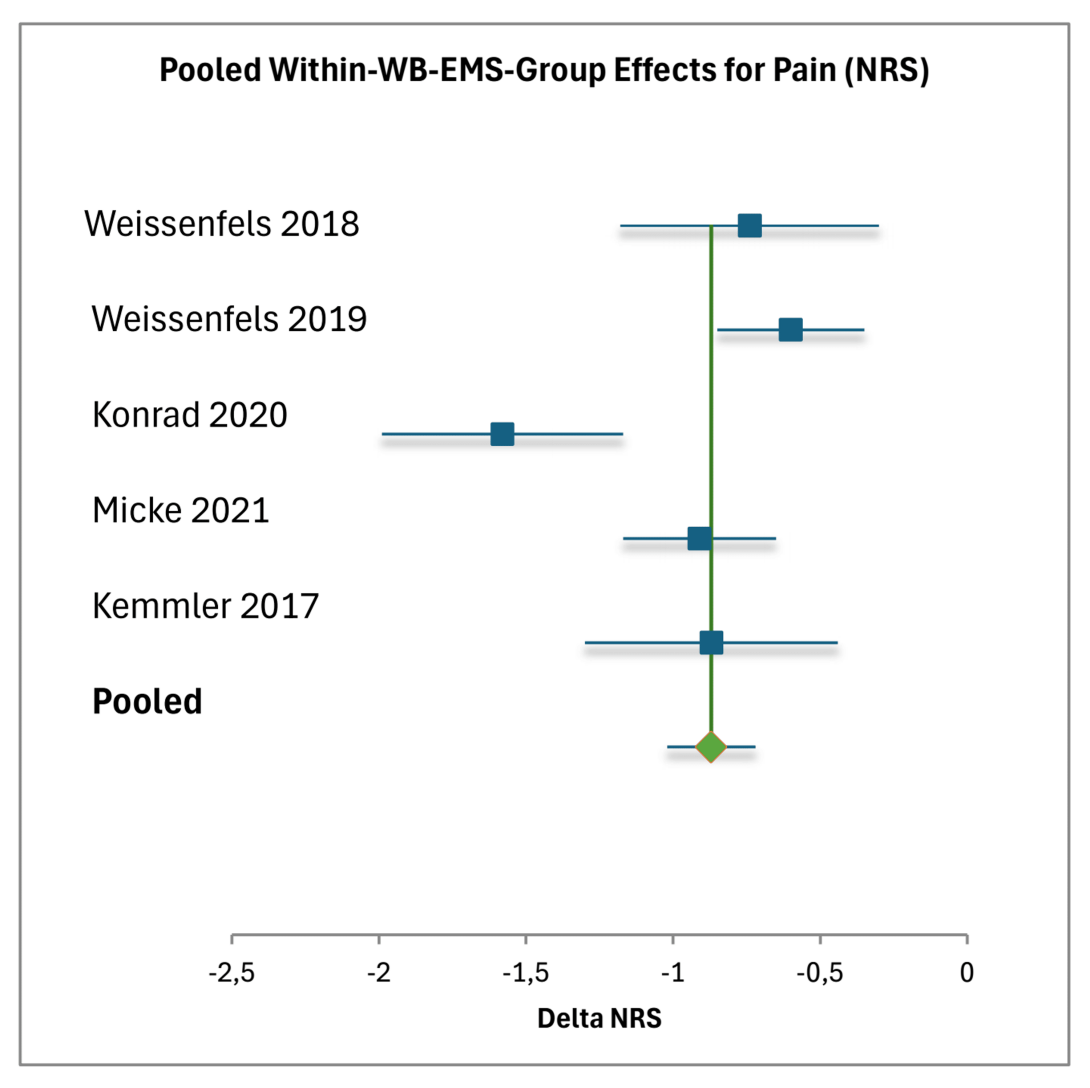
Forest plot of pooled within-WB-EMS group effects for pain (NRS) Individual effects are shown for Weissenfels et al. (2018) [17], Weissenfels et al. (2019) [18], Konrad et al. (2020) [19], Micke et al. (2021) [20], and Kemmler et al. (2017) [21], with the pooled effect as a diamond. Horizontal lines represent 95% CIs; the vertical line at 0 indicates no effect. Negative values reflect pain reduction. Heterogeneity: $I^2$ = 70% (random-effects model) [34]. WB-EMS: whole-body electromyostimulation; NRS: Numeric Rating Scale

**Figure 4:**
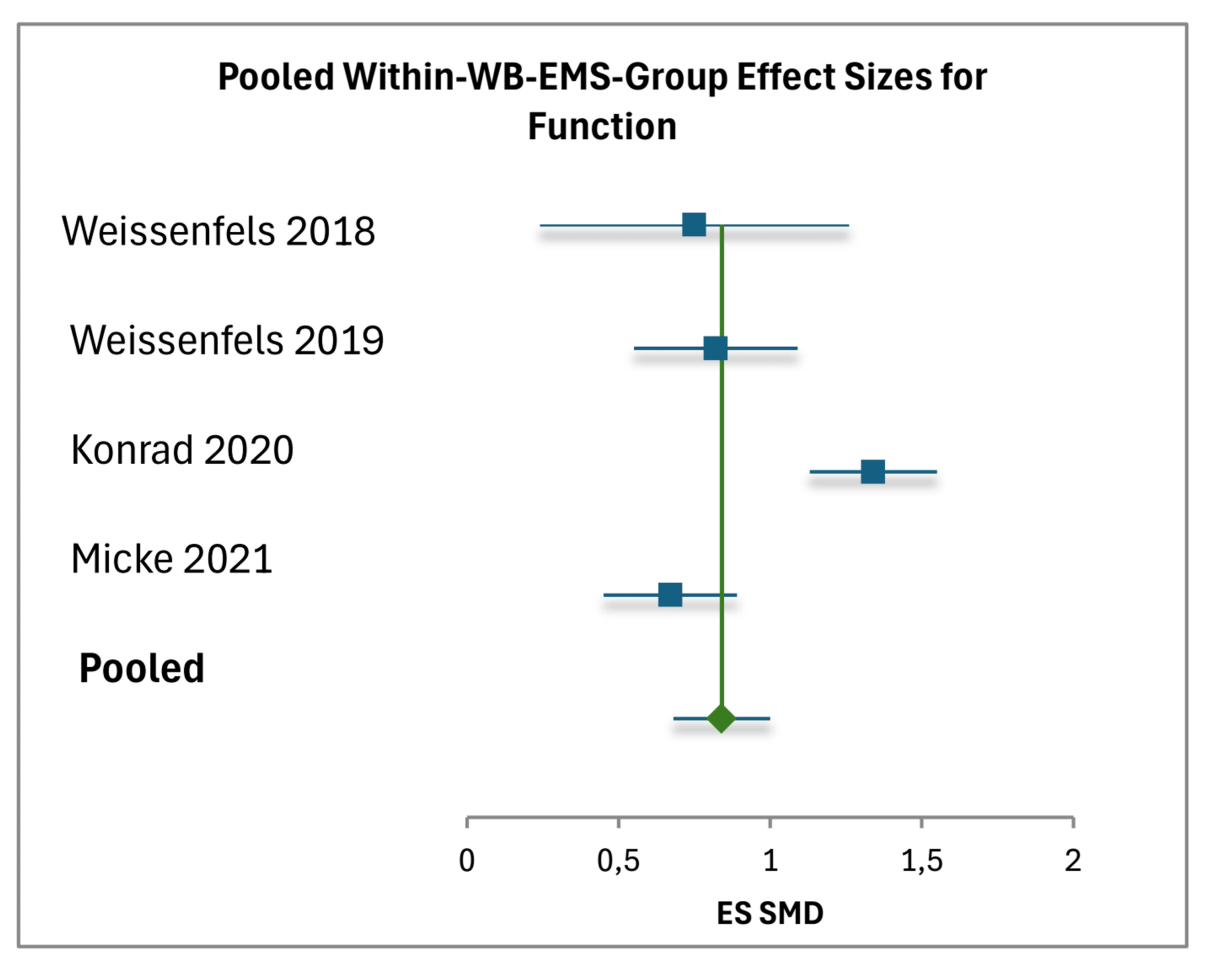
Forest Plot of Pooled Within-WB-EMS Group Effect Sizes for Function Individual effects are shown for Weissenfels et al. (2018) [17], Weissenfels et al. (2019) [18], Konrad et al. (2020) [19], and Micke et al. (2021) [20], with the pooled effect as a diamond. Horizontal lines represent 95% CIs; the vertical line at 0 indicates no effect. Positive values reflect functional improvement. Heterogeneity: $I^2$ = 76% (random-effects model) [34]. WB-EMS: whole-body electromyostimulation

The meta-analysis examined the within-group effects of WB-EMS as well as the comparative effects against PCG and ACG. For the within-group analysis, data were extracted from five studies, with sample sizes ranging from 15 to 85 participants, totaling 258 participants [17-21]. The pooled within-group effect size for pain reduction (measured on the NRS (0-10 scale)) was -0.87 (95% CI (-1.02, -0.72)), based on a total weight of 178.83, with individual study effect sizes ranging from -0.60 to -1.58 [17-21]. Weights were calculated as $Weight = \frac{1}{Variance}$, where $Variance = \frac{SD^2}{N}$, and the pooled effect was derived using $Pooled\ Effect = \frac{\sum (Weight \times Effect)}{\sum Weight}$. The standard error for the pooled pain effect was 0.0673, and heterogeneity was moderate with an I² of 70%. For functional outcomes, reported in varying units (e.g., kilograms for trunk strength, ODI for disability), the pooled SMD was 0.84 (95% CI (0.68, 0.99)), with a total weight of 158.36 and a standard error of 0.079. Individual function effect sizes ranged from 0.67 to 1.34, with moderate heterogeneity (I²=76%) [17-21]. Negative delta values indicating improvement (e.g., -15.8 ODI) were adjusted to positive values for consistency [19] (Figures 3, 4).

In the comparative analysis, the WB-EMS group was compared to a PCG (no intervention) and an ACG (e.g., back-strengthening training, multimodal treatment, or whole-body vibration). For WB-EMS versus PCG, based on a sample size of 15, the pooled effect size for pain was 0.75 (95% CI (0.01, 1.50)), with a weight of 7.00, and for function, it was 0.85 (95% CI (0.11, 1.60)), with a weight of 6.87; heterogeneity was not applicable due to a single study (Table 3) [17].

**Table 3:** ES for pain and function: WB-EMS vs. PCG Pain and function ES (SMD) with 95% CIs and weights are reported for Weissenfels et al.,2018 [17] (PCG: no intervention). Positive values indicate WB-EMS superiority. Pooled SMD: 0.75 (95% CI (0.01, 1.50), weight 7.00) for pain; 0.85 (95% CI (0.11, 1.60), weight 6.87) for function. Heterogeneity not applicable (single study) [34]. ES: effect sizes; SMD: standardized mean differences; WB-EMS: whole-body electromyostimulation; PCG: passive control group

Study	Pain ES (SMD)	95% CI	Weight	Function ES (SMD)	95% CI	Weight
Weissenfels et al., 2018 [17]	0.75	[0.01, 1.50]	7	0.85	[0.11, 1.60]	6.87
Pooled	0.75	[0.01, 1.50]	7	0.85	[0.11, 1.60]	6.87

**Figure 5:**
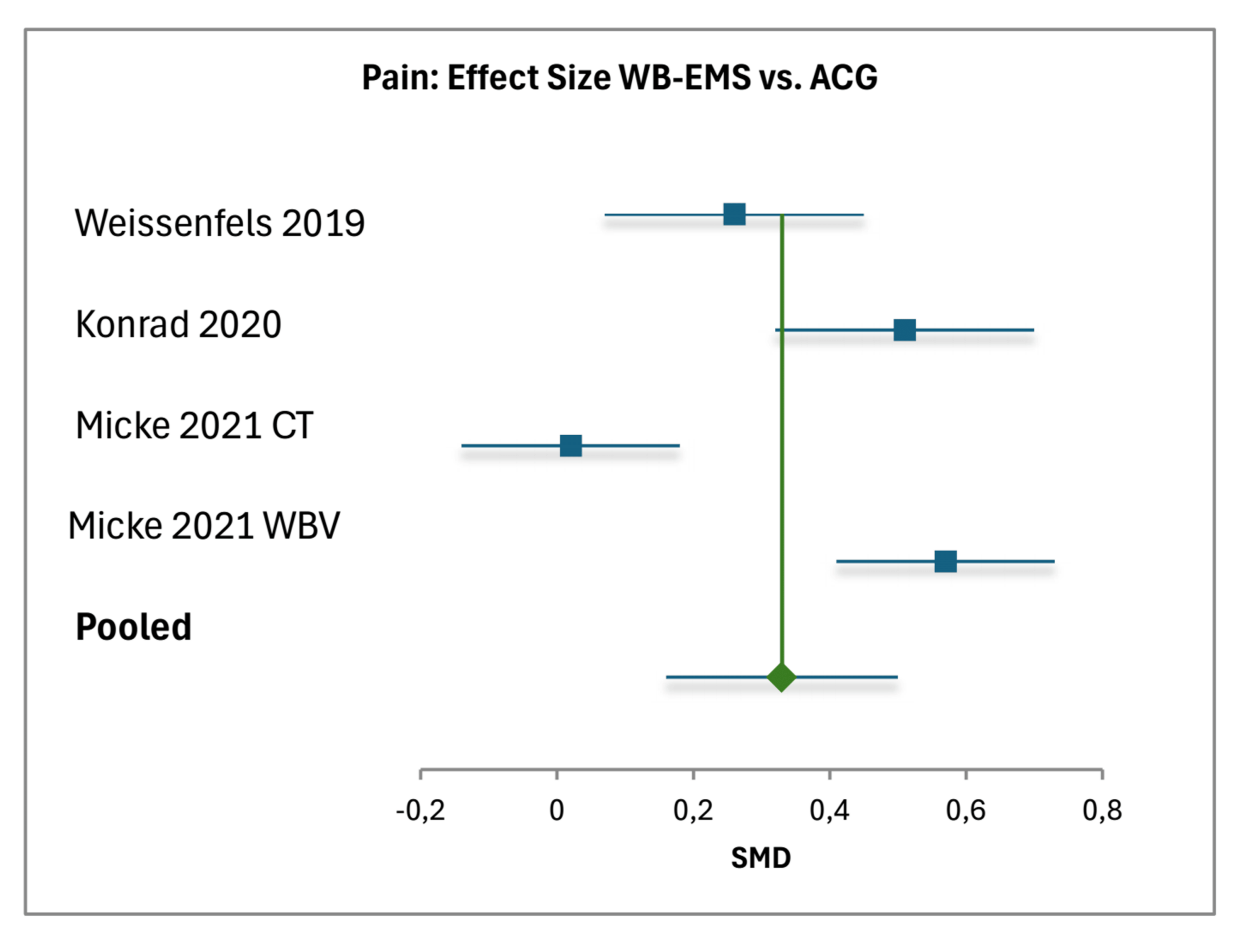
Forest plot of effect sizes for pain: WB-EMS vs. ACG Individual effects are shown for Weissenfels et al. (2019) [18] (ACG: back-strengthening training), Konrad et al., (2020) [19] (ACG: multimodal treatment), and Micke et al. (2021) [20] (ACG: CT, WBV), with the pooled effect as a diamond. Horizontal lines represent 95% CIs; the vertical line at 0 indicates no difference. Positive values favor WB-EMS. Pooled SMD: 0.33 (95% CI (0.16, 0.50), weight 133.41). Heterogeneity: $I^2$ = 96% (random-effects model) [34]. WB-EMS: whole-body electromyostimulation; ACG: active control group; CT: conventional training: SMD: standardized mean differences

**Figure 6:**
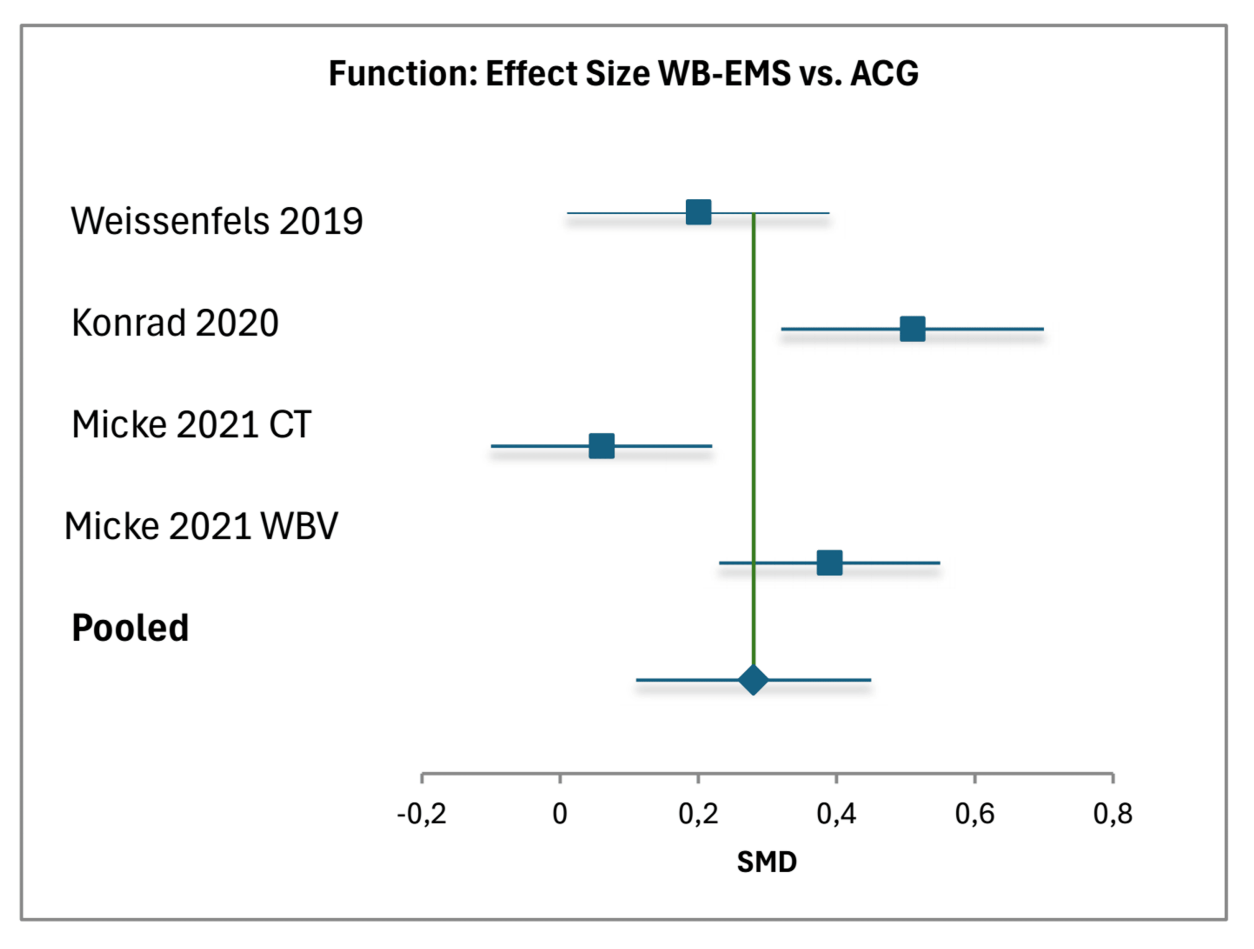
Forest Plot of Effect Sizes for Function: WB-EMS vs. ACG Individual effects are shown for Weissenfels et al. (2019) [18] (ACG: back-strengthening training), Konrad et al., (2020) [19] (ACG: multimodal treatment), and Micke et al. (2021) [20] (ACG: CT, WBV), with the pooled effect as a diamond. Horizontal lines represent 95% CIs; the vertical line at 0 indicates no difference. Positive values favor WB-EMS. Pooled SMD: 0.28 (95% CI [0.11, 0.45], weight 134.24). Heterogeneity: $I^2$ = 92% (random-effects model) [34]. WB-EMS: whole-body electromyostimulation; ACG: active control group; CT: conventional training: SMD: standardized mean differences

For WB-EMS versus ACG, data from three studies (including CT and WBV subgroups, total N=343) yielded a pooled pain effect size of 0.33 (95% CI (0.16, 0.50)), based on a total weight of 133.41, with individual effect sizes ranging from 0.02 to 0.57 [18-20]. The standard error was 0.0866, with high heterogeneity (I²=96%). The pooled function effect size was 0.28 (95% CI (0.11, 0.45)), with a total weight of 134.24 and a standard error of 0.0863, ranging from 0.06 to 0.51, with high heterogeneity (I²=92%) [18-20]. Forest plots visually confirmed these findings, illustrating the effect sizes and confidence intervals for each comparison (Figures 5, 6).

Discussion

Overview of Findings

The narrative synthesis and meta-analysis collectively suggest preliminary evidence for the efficacy of WB-EMS in managing NSCBP, with consistent findings across both qualitative and quantitative analyses. Within-group improvements in pain, ranging from -0.60 to -1.58 NRS/VAS points, and function, ranging from +7.19 kg trunk strength to -15.8 ODI, were observed across all five studies. These improvements are supported by the meta-analytic pooled estimates, which showed a pain reduction of -0.87 NRS points (95% CI (-1.02, -0.72)) and a functional SMD of 0.84 (95% CI (0.68, 0.99)).

The moderate heterogeneity in the within-group analysis (I² = 70% for pain, I² = 69% for function) suggests variability across studies, which may be attributed to differences in intervention protocols (e.g., duration, frequency, intensity), participant characteristics (e.g., age, baseline pain severity), and outcome measures (e.g., NRS, VAS, ODI, trunk strength). For instance, Konrad et al. (2020) reported the largest pain reduction (-1.58 NRS points), potentially due to a higher baseline pain level, while Weissenfels et al. (2019) showed a smaller reduction (-0.60 NRS points), possibly reflecting a less severe cohort [18,19]. This hypothesis is supported by the study of Konrad et al. (2024), where a positive influence of baseline pain intensity on pain reduction has been shown [33].

Comparative Performance

WB-EMS demonstrated a clear advantage over PCGs, with effect sizes of 0.75 for pain and 0.85 for function in Weissenfels et al. (2018) [17], suggesting a strong benefit over no intervention. These findings align with the broader literature on physical interventions for NSCBP, where active treatments consistently outperform no treatment in reducing pain and improving function (Hayden et al., 2005; Searle et al., 2015) [11,12].

Against ACGs, WB-EMS showed smaller but still significant advantages, with pooled effect sizes of 0.33 for pain (95% CI (0.16, 0.50)) and 0.28 for function (95% CI (0.11, 0.45)). However, the high heterogeneity in the ACG analysis (I² = 96% for pain, I² = 92% for function) indicates substantial variability, likely driven by the diverse nature of the active comparators, which ranged from back-strengthening training (Weissenfels et al., 2019) to multimodal treatment (Konrad et al., 2020) and WBV or conventional training (Micke et al., 2021) [18-20]. For example, Konrad et al.’s (2020) multimodal treatment, involving a whole-day intervention five times per week, contrasts sharply with the time-efficient 20-minute WB-EMS sessions, potentially influencing the observed effect sizes. This variability underscores the need for standardized comparator interventions in future studies to better isolate the specific effects of WB-EMS.

Methodological Insights

The exclusion of Silvestri et al. (2023) from the comparative meta-analysis, due to the absence of a control group without WB-EMS, and Kemmler et al. (2017) from PCG/ACG analysis, due to its mixed control group, limits the comparative insights but strengthens the within-group evidence [21,22].

Silvestri et al. (2023) suggest that combining WB-EMS with stretching may enhance outcomes, as the WB-EMS + stretching group showed a greater pain reduction (-2.76 VAS points) and functional improvement (-7.42 ODI-I) compared to WB-EMS alone (-0.935 VAS, -1.53 ODI-I) [22]. However, its low PEDro score (4/10) due to non-randomization and lack of blinding warrants cautious interpretation.

Similarly, Kemmler et al.’s (2017) within-group data (-0.87 NRS) contribute to the overall evidence of WB-EMS efficacy, but its mixed control group precluded its inclusion in the comparative analysis, highlighting the importance of clearly defined control groups [21].

Methodological quality, as assessed by PEDro scores, varied from four to eight, with RCTs like Weissenfels et al. (2018, 2019) and Micke et al. (2021) scoring 8/10, reflecting strengths in randomization, concealed allocation, and follow-up [17,18,20]. Konrad et al. (2020) scored 6/10 due to its non-randomized design, a limitation justified by the impracticality of blinding and randomizing between multimodal treatment and WB-EMS [19]. Silvestri et al.’s (2023) score of 4/10 further underscores the need for higher-quality studies to confirm additive effects like stretching [22]. The lack of blinding in most studies, particularly in Konrad et al. (2020), may introduce performance bias, though self-reported outcomes (e.g., NRS, ODI) mitigate detection bias to some extent.

The adjustment of scales, converting Kemmler et al.’s (2017) 0-7 scale to NRS by multiplying by 10/7 and deeming Silvestri et al.’s (2023) 0-10 VAS equivalent to NRS, facilitated consistency but introduced potential measurement error, as VAS and NRS, despite similar ranges, differ in their continuous versus categorical nature [21,22].

Clinical Relevance and Minimal Clinically Important Difference (MCID)

The clinical relevance of the observed pain reductions with WB-EMS can be evaluated by comparing the results to established thresholds for the MCID, which represents the smallest change in pain intensity perceived as meaningful by patients. In this meta-analysis, the pooled within-group pain reduction was -0.87 NRS points (95% CI (-1.02, -0.72)), with individual study effects ranging from -0.60 to -1.58 NRS points [17-21].

Studies on MCID for chronic back pain provide context for interpreting these findings. Bråten et al. (2022), Haase and Kladny (2021), Salaffi et al. (2004), and Ostelo et al. (2008) collectively suggest an MCID for NRS in chronic back pain ranging from one to two points or a 15%-33% reduction, with recent evidence supporting a 15%-20% threshold for moderate baseline pain levels (e.g., NRS 4-6) [37-40]. In line with current trends, Bråten et al. (2022) and Haase and Kladny (2021) indicate that a 15% reduction may be clinically meaningful, aligning with the observed -0.87 NRS reduction, whereas earlier studies by Farrar et al. (2001) and Dworkin et al. (2008) propose a higher 30% threshold for robust changes [41,42]. The pooled -0.87 NRS reduction in our study is just below the one-point MCID threshold for "slightly better" improvement but aligns with the lower end of the percentage-based MCID (15%-20% reduction from typical baseline NRS scores of four to six) [37-42].

However, individual study effects, such as the -1.58 NRS reduction in Konrad et al. (2020) [19], exceed this threshold, indicating potential relevance for some patients. This variability is further supported by Konrad et al. (2024), which stratified pain reduction by baseline pain intensity, finding a moderate positive correlation (r_s = 0.551, p < 0.001) between baseline NRS and pain reduction, with the greatest improvements (3.72 NRS points) in patients with severe baseline pain (NRS > 7) [33]. While Konrad et al. (2020) [19] and Konrad et al. (2024) [33] report the percentage of participants reaching an MCID threshold of ≥2 points, this criterion is considered outdated in light of newer evidence favoring 15%-20% reductions; thus, current data lack comprehensive reporting of participants achieving the updated MCID, which should be a focus of future research to enhance clinical applicability.

These findings suggest that while the average pain reduction with WB-EMS may be on the lower end of MCID thresholds, specific subgroups, particularly those with moderate to severe baseline pain, achieve clinically meaningful improvements, highlighting the need for future studies to stratify outcomes by baseline pain severity.

Practical Implications

Clinically, WB-EMS’s time efficiency (20-minute sessions twice weekly) makes it a practical alternative to more resource-intensive interventions like multimodal treatment, as seen in Konrad et al. (2020), where WB-EMS achieved comparable outcomes (-1.58 NRS, -15.8 ODI) to a whole-day program (+0.66 NRS, -0.88 ODI) [19]. This is particularly relevant for NSCBP patients with time constraints or mobility limitations, as highlighted in the introduction [13,14].

The safety profile of WB-EMS is also noteworthy, with adverse events limited to mild muscle soreness in one to five participants per study, supporting its suitability for patients with kinesiophobia or joint impairments [17-22].

However, the intervention durations (eight to 16 weeks) limit insights into long-term efficacy, and the small number of studies (n=6 for within-group, n=4 for comparative) and sample sizes (e.g., Weissenfels et al., 2018: n=30) [17] restrict generalizability. Dropout rates, such as 23% in Silvestri et al. (2023) [22], further suggest potential challenges in adherence, which future studies should address through patient-centered protocols.

Future Research Directions

The large SMD (>0.8) for within-group function aligns with prior WB-EMS literature (e.g., Kemmler et al., 2017) [21], indicating a clinically meaningful impact on functional outcomes like trunk strength and disability. However, the smaller comparative effect sizes against ACG (0.33 for pain, 0.28 for function) suggest that while WB-EMS is at least as effective as active interventions, it may not offer a marked superiority in all contexts. This equivalence is valuable, given WB-EMS’s efficiency and accessibility, but the high heterogeneity in ACG comparisons calls for further research to identify which active interventions are most comparable and under what conditions WB-EMS might outperform them [18-20].

A notable limitation in the comparative analysis is the reliance on a single study (Weissenfels et al., 2018) with only n=15 participants per group for the evaluation of WB-EMS versus PCGs [17]. This small sample size restricts statistical power and generalizability, potentially inflating perceived benefits due to random variation or selection bias. The absence of additional studies with passive controls further underscores the need for more research to confirm these effects.

Additionally, the current evidence base is limited to LF-WB-EMS, as all studies on WB-EMS and NSCBP published in PubMed to date have exclusively investigated LF-EMS (0-999 Hz). There is a notable lack of research on MF-WB-EMS (1-300 kHz), which is hypothesized to offer more physiological contractions and deeper tissue penetration. Future studies investigating MF-WB-EMS, both in isolation and in direct comparison to LF-WB-EMS, are essential to determine whether frequency variations influence treatment outcomes and to optimize EMS protocols for NSCBP.

Future research should prioritize longer-term follow-ups beyond 16 weeks to assess the sustainability of WB-EMS benefits, particularly for functional outcomes, which may require ongoing intervention to maintain gains [17-22].

Standardizing outcome measures (e.g., using NRS for pain and ODI for function across studies) would enhance comparability and facilitate more robust meta-analyses.

Larger, RCTs with clearly defined control groups are needed to confirm synergistic effects, such as the addition of stretching observed in Silvestri et al. (2023) [22], and to refine optimal protocols (e.g., frequency, duration, intensity).

Investigating patient-specific factors, such as age, gender, pain chronicity, or comorbidities like osteoarthritis, could further tailor WB-EMS application in clinical practice, ensuring personalized treatment plans that maximize efficacy and adherence.

Furthermore, reporting the percentage of participants reaching MCID-level improvement would enhance the clinical applicability. Recent studies on MCID also indicate that the percentage improvement from baseline is a better metric for assessing clinical significance than mean pain delta. Therefore, it would be valuable for future studies to provide the average relative pain improvement from baseline for a better evaluation of WB-EMS’s effectiveness [37-39]. Given the high heterogeneity (I² > 90% for ACG comparisons), future research should consider subgroup analyses or meta-regressions to explore sources such as intervention duration, frequency, intensity, and participant characteristics, which may contribute to the observed variability.

## Conclusions

Overall, the results indicate that LF-WB-EMS can reduce pain and improve function in NSCBP, offering possible benefits based on within-group trends and competitive performance against controls. The within-group effects (-0.87 NRS, 0.84 SMD) and comparative effects (0.75/0.85 vs. PCG, 0.33/0.28 vs. ACG) suggest potential advantages, though the superiority over other therapy methods remains uncertain due to high heterogeneity, modest effect sizes, and variable study quality. Despite these limitations, WB-EMS demonstrates at least comparable efficacy to established methods in this investigation, positioning it as a promising alternative, particularly for patients with time constraints or mobility limitations, supported by its time efficiency and joint-friendly nature. Its safety profile, marked by minimal adverse events, further supports its applicability for patients with mobility issues. However, the reliance on a single small PCG study (n=15) and significant heterogeneity in active control comparisons underscore the need for larger, standardized trials. Additionally, the lack of research on medium-frequency WB-EMS and direct comparisons with LF-EMS highlights the need for further investigation to refine protocols and clarify long-term efficacy.

## Appendices

Appendix A

Detailed PubMed Search Query

Search: (("whole-body electromyostimulation") OR (WB-EMS)) AND ((back pain) OR (chronic back pain) OR (nonspecific back pain))

("whole-body electromyostimulation"[All Fields] OR "WB-EMS"[All Fields]) AND ("back pain"[MeSH Terms] OR ("back"[All Fields] AND "pain"[All Fields]) OR "back pain"[All Fields] OR (("chronic"[All Fields] OR "chronical"[All Fields] OR "chronically"[All Fields] OR "chronicities"[All Fields] OR "chronicity"[All Fields] OR "chronicization"[All Fields] OR "chronics"[All Fields]) AND ("back pain"[MeSH Terms] OR ("back"[All Fields] AND "pain"[All Fields]) OR "back pain"[All Fields])) OR (("nonspecific"[All Fields] OR "nonspecifically"[All Fields] OR "nonspecificity"[All Fields]) AND ("back pain"[MeSH Terms] OR ("back"[All Fields] AND "pain"[All Fields]) OR "back pain"[All Fields])))

Translations: back pain: "back pain"[MeSH Terms] OR ("back"[All Fields] AND "pain"[All Fields]) OR "back pain"[All Fields]

chronic: "chronic"[All Fields] OR "chronical"[All Fields] OR "chronically"[All Fields] OR "chronicities"[All Fields] OR "chronicity"[All Fields] OR "chronicization"[All Fields] OR "chronics"[All Fields]

back pain: "back pain"[MeSH Terms] OR ("back"[All Fields] AND "pain"[All Fields]) OR "back pain"[All Fields]

nonspecific: "nonspecific"[All Fields] OR "nonspecifically"[All Fields] OR "nonspecificity"[All Fields]

back pain: "back pain"[MeSH Terms] OR ("back"[All Fields] AND "pain"[All Fields]) OR "back pain"[All Fields]
